# Notes and News

**Published:** 1897-02-27

**Authors:** 


### NOTES AND NEWS.

(Collected and Communicated.)

### HOSPITAL MEETINGS.

University College Hospital, Gower Street.—Annual general meeting, Friday, March 19th, four p.m., in Board-room of the Hospital.

National Orthopædic Hospital, 234, Great Portland Street, W.—Annual meeting of governors, Friday, February 26th, four p.m., at the Hospital.

The Hospital for Women, 144, Euston Road.—Annual meeting of governors at the Hospital, Thursday, March 4th, half-past three.

The Indian Famine Fund now amounts to £344,000.

The Queen has sent a donation of £10 to the Royal National Hospital for Consumption at Ventnor.

The Barker anatomical prizes, in connection with the Royal College of Surgeons of Ireland, are open to students whose name is on the anatomical class list of any medical school in the United Kingdom. The prize now offered is of the value of £26 5s., and will be awarded for a dissection of the region of the sterno mastoid; above the crioid, with special relation to lateral pharyngotomy; the removal of tonsillar tumours and of infiltrated glands in cancer of the tongue. Full particulars can be obtained from Dr. Scott, curator of the museum.

An interesting ceremony took place last week at St. Mary's Hospital, when Sir William Broadbent was presented with some beautiful plate as a mark of appreciation of his services on his retirement as senior physician to the hospital. The plate, which included a variety of works of art in the form of candelabra, bowl and tea tray, and other articles, was presented by the staff of the hospital and students who had studied under Sir William Broadbent. The gifts were accompanied by an album containing the names of the donors and a complimentary inscription. Sir William Broadbent, in accepting the presentation, alluded to the development of the hospital since he had first known it, and expressed a hope that the wish near his heart that the institution would one day include special wards, nursing home, and medical college, all concentrated upon one spot, would be realised.

We learn that Sir Blundell Maple has promised the magnificent sum of £100,000 for the purpose of rebuilding University College Hospital, as a commemoration of the sixtieth year of the Queen's reign.

A serious outbreak of cholera at Rewa in India is reported. It is stated that 160 deaths occurred in two days, and that energetic steps are being taken to check the progress of the disease.

Dr. Koch's investigations relating to rinderpest have been carried on with great zeal since his arrival at the Cape. He is himself most hopeful of establishing a process of immunisation, and has further been able to throw very considerable light upon the subject of the disease which cannot fail to be of value.

Last week the Duke and Duchess of York opened the Dartmouth Home for Crippled Boys, at Blackheath. The Home is to serve as a technical school to the parent institution at Wimbledon. The site of the new Home was presented by the Earl of Dartmouth, hence its name. After the opening ceremony the Duchess of York received purses in aid of the charity.

The Birmingham Board of Health recently invited the Board of Guardians to a conference on the subject of the anti-toxin treatment of diphtheria. It appears that the percentage of deaths from diphtheria at Birmingham has been very large up to the present time, and that no systematic application of the anti-toxin treatment has been resorted to. As a result of the conference, the Guardians express themselves disposed to adopt the suggestion of the Health Committee that syringes and serum shall be supplied to the district medical officers. The use of the serum seems to have been little resorted to generally in Birmingham, and this also the Board of Health desire to see altered.

The Sanitary Conference, which is being held in Venice, is proceeding with its deliberations in an apparently friendly spirit. Turkey still remains the difficulty. Whatever steps are taken will probably follow the lines of the Paris Convention, which unfortunately is still unratified by the Porte. Dr. Cleghorn, representing the Indian Government, has arrived; and, as showing the anxiety felt in Italy in regard to the possible introduction of the plague, it may be mentioned that he was the only passenger who was allowed to land at Brindisi, all the rest being compelled to proceed to Plymouth by sea.

The plague continues unabated at Bombay, the official record given on February 5th for the preceding day being 116 cases and 93 death; but, as we pointed out last week, the real mortality probably far exceeds that which is officially recorded. The *Times of India* for February 5th gives some interesting details as to the death-rates now prevalent among the various races and castes in Bombay. The highest death-rate (from all causes) is among the low-caste Hindoos, being at the rate of 164 per 1,000. Then come Hindoos and other castes, 110 per 1,000; Mohammedans, 86 per 1,000; native Christians, 147 per 1,000. Even the Brahmins are dying at the rate of 71 per 1,000. Meanwhile, the death-rate among the Europeans is only 23 per 1,000, or less than one-third the mortality in any other class. Comparisons, although odious, are instructive, and to show what is going on, it may be as well to state that the general death-rate of Bombay was five times as great, and that of certain castes eight times as great, as that prevailing in London at the same time.

